# Crystal structure of (3*R*,5a*S*,6*R*,9*R*,12*R*,12a*R*)-3,6,9-tri­methyl­deca­hydro-12*H*-3,12-ep­oxy[1,2]dioxepino[4,3-*i*]isochromen-10-yl 5-((3a*S*,4*S*,6a*R*)-2-oxohexa­hydro-1*H*-thieno[3,4-*d*]imidazol-4-yl)penta­noate

**DOI:** 10.1107/S2056989026004895

**Published:** 2026-05-29

**Authors:** Xiaomei Yang, Siyu Chen, Wenxin Liu, Mengjie Cui, Qingjie Zhao

**Affiliations:** a Innovation Research Institute of Traditional Chinese Medicine Shanghai University of Traditional Chinese Medicine,Shanghai 201203 People’s Republic of China; b College of Life Sciences, Shanghai Normal University,Shanghai 201418, People’s Republic of China; University of Aberdeen, United Kingdom

**Keywords:** crystal structure, di­hydro­artemisinin, biotin conjugate, endoperoxide bridge, anti­tumor activity

## Abstract

In the title biotin-conjugated di­hydro­artemisinin (DHA) derivative, C_25_H_38_N_2_O_7_S, the mol­ecule retains the essential endoperoxide bridge and links the C-10 position of DHA to the penta­noate chain of biotin *via* an ester bond. In the solid state, the tetra­hydro­pyran ring of DHA adopts a chair conformation, the fused peroxide seven-membered ring exhibits a twist conformation, the imidazolidone ring of biotin shows an envelope conformation and the tetra­hydro­thio­phene ring adopts a twisted conformation. Supra­molecularly, adjacent mol­ecules are linked through a classical N—H⋯O double hydrogen-bonding motif between the urea groups of biotin, forming anti­parallel cyclic dimers. These strong dimers are further supported by weaker C—H⋯O inter­actions. The SQUEEZE [Spek (2015 ▸). *Acta Cryst*. C**71**, 9-18] routine in *PLATON* was used to remove electron density corresponding to disordered solvent mol­ecules. This structure determination provides a valuable blueprint for the rational design of hybrid anti­malarial and anti­cancer therapies based on DHA–biotin conjugates.

## Abstract 2

In the title biotin-conjugated di­hydro­artemisinin (DHA) derivative, the mol­ecule retains the essential endoperoxide bridge and links the C-10 position of DHA to the penta­noate chain of biotin *via* an ester bond. This structure determination provides a valuable blueprint for the rational design of hybrid anti­malarial and anti­cancer therapies based on DHA–biotin conjugates.

## 1. Chemical context

Artemisinin (C_15_H_22_O_5_; ART), isolated from *Artemisia annua*, and its active metabolite di­hydro­artemisinin (C_15_H_24_O_5_; DHA) are cornerstone anti­malarial agents, particularly effective against drug-resistant *Plasmodium falciparum* strains. Their pharmacological significance extends to oncology, where DHA exhibits potent anti­tumor activity across various cancers, including breast, lung, and melanoma, by inhibiting angiogenesis, inducing apoptosis, and promoting ferroptosis through iron-dependent reactive oxygen species (ROS) generation. The endoperoxide bridge in ART and DHA is crucial for cytotoxicity. In the presence of ferrous iron, this moiety undergoes homolytic cleavage, yielding carbon-centered radicals that alkyl­ate biomacromolecules and trigger oxidative stress, leading to parasite and cancer cell death. Derivatives lacking this bridge, such as de­oxy­artemisinin, show markedly reduced potency, underscoring its essential role.

Conjugating DHA with biotin – a vitamin overexpressed on tumor cells *via* specific transporters – offers significant biological advantages. Biotinylation enhances tumor targeting, improves cellular uptake, and enables avidin-mediated delivery systems, boosting efficacy while minimizing off-target effects, as demonstrated in biotin-ART micelle formulations that reduced tumor volumes in breast cancer models.
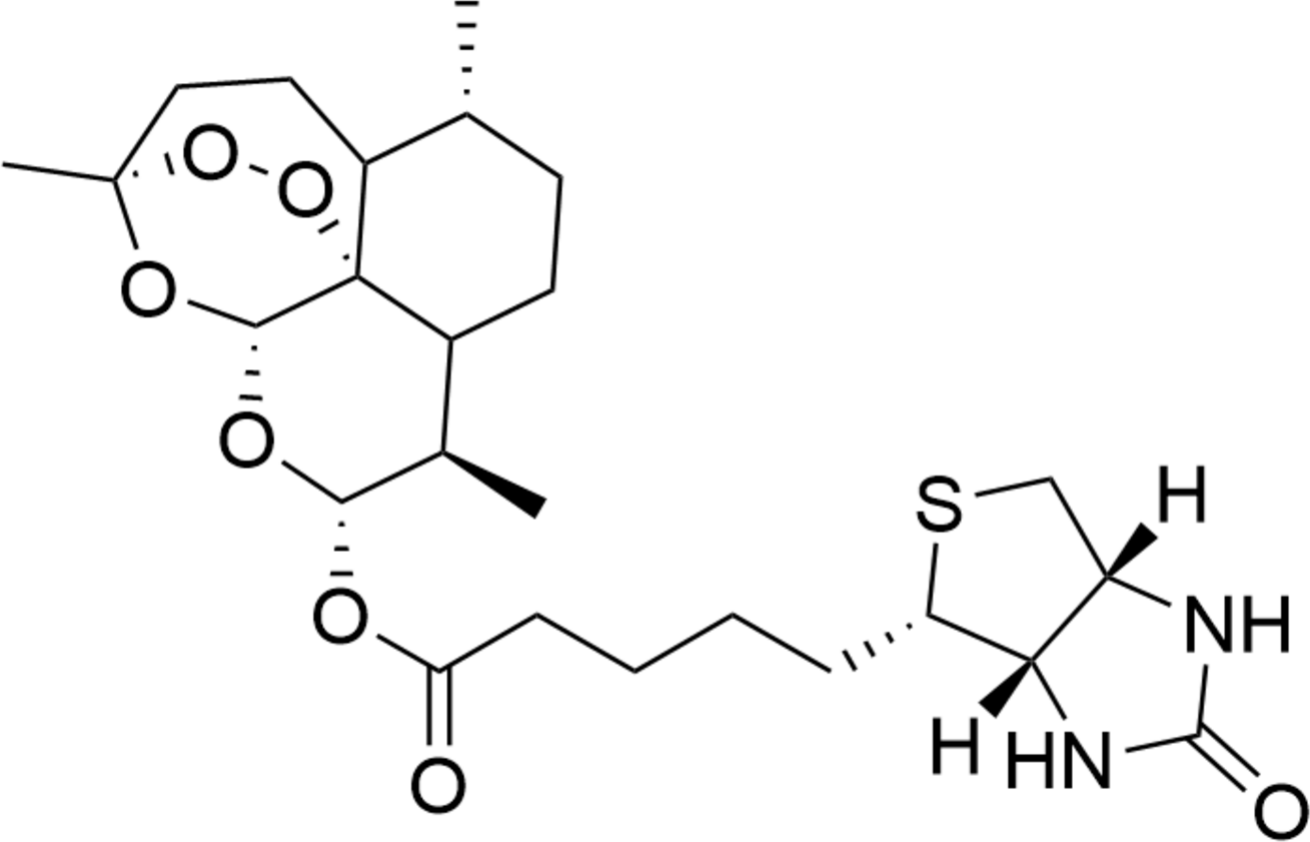


As part of our studies in this area, we now report the synthesis and single-crystal structure of the title biotin-conjugated DHA derivative, C_26_H_40_N_2_O_6_S (**I**). This polycyclic scaffold preserves the peroxide bridge while linking DHA’s C-10 atom to biotin’s penta­noate chain, potentially optimizing pharmacokinetics and selectivity.

## 2. Structural commentary

**Figure 1:**
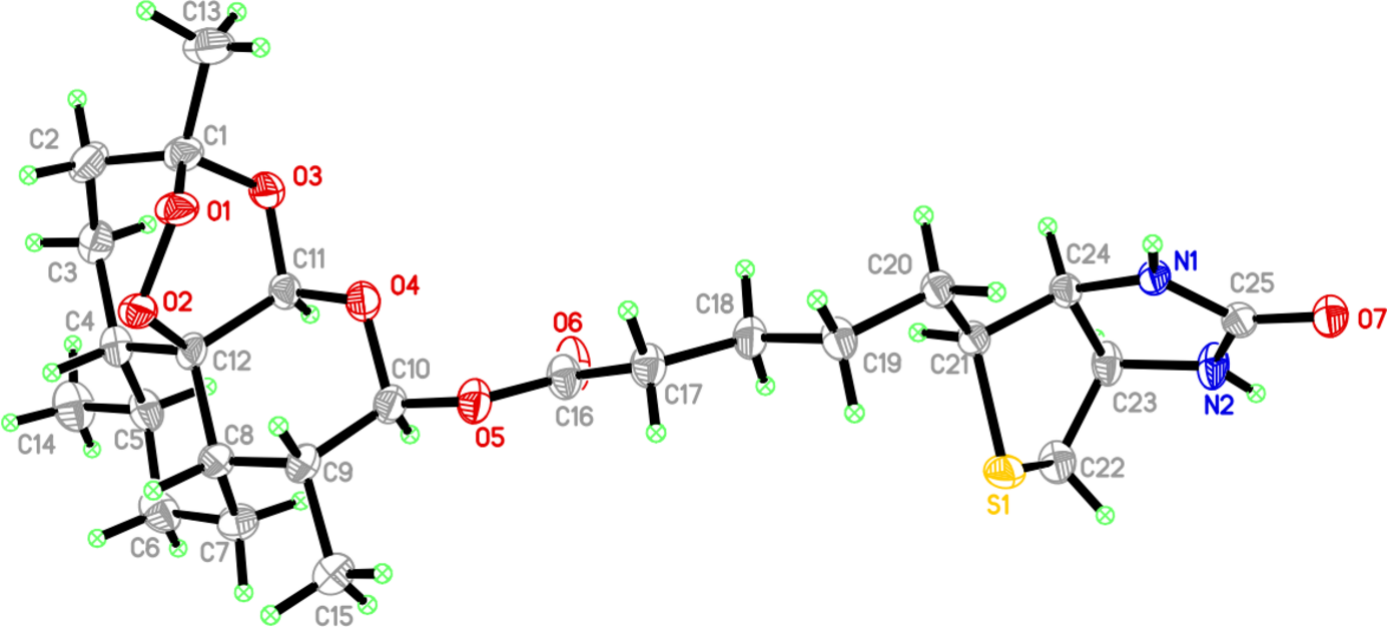
The mol­ecular structure of (**I**) showing 50% probability ellipsoids.

The crystal of (**I**) belongs to the monoclinic system, space group *P*2_1_, with *Z* = 2, containing one complete mol­ecule in the asymmetric unit (Fig. 1 ▸). Mol­ecule (**I**) consists of a di­hydro­artemisinin (DHA) core linked to a biotin side chain through an ester bond. The DHA moiety retains its natural absolute configuration of (3*R*, 5a*S*, 6*R*, 9*R*, 12*R*, 12a*R*), while the biotin moiety adopts a (3a*S*, 4*S*, 6a*R*) configuration. Key bond lengths are as follows: the per­oxy bridge O1—O2 separation is 1.462 (4) Å, the ester C10—O5 bond is 1.416 (4) Å, and the carbonyl C16=O6 bond is 1.206 (5) Å, all within normal ranges. The crucial O5—C10—O4 bond angle measures 104.3 (3)°. Four representative torsion angles are C1—O1—O2—C12 = 46.5 (4), C8—C9—C10—O5 = 178.5 (3), C11—O4—C10—O5 = 1778.0 (3) and C15—C9—C10—O5 = −56.8 (5)°. The tetra­hydro­pyran (C4–C8/C12) ring in the DHA core adopts a stable chair conformation, while the fused peroxide and seven-membered ring (C1–C4/C12/C11/O3) exhibits a twist conformation. The imidazolidone ring in the biotin unit (N1/C24/C23/N2/C25) displays an envelope conformation, and the tetra­hydro­thio­phene ring (S1/C21/C24/C23/C22) shows a twisted conformation. The overall stereochemistry of the mol­ecule agrees with the expected configuration, and formation of the ester linkage does not introduce any significant conformational distortion.

## 3. Supra­molecular features

**Figure 2:**
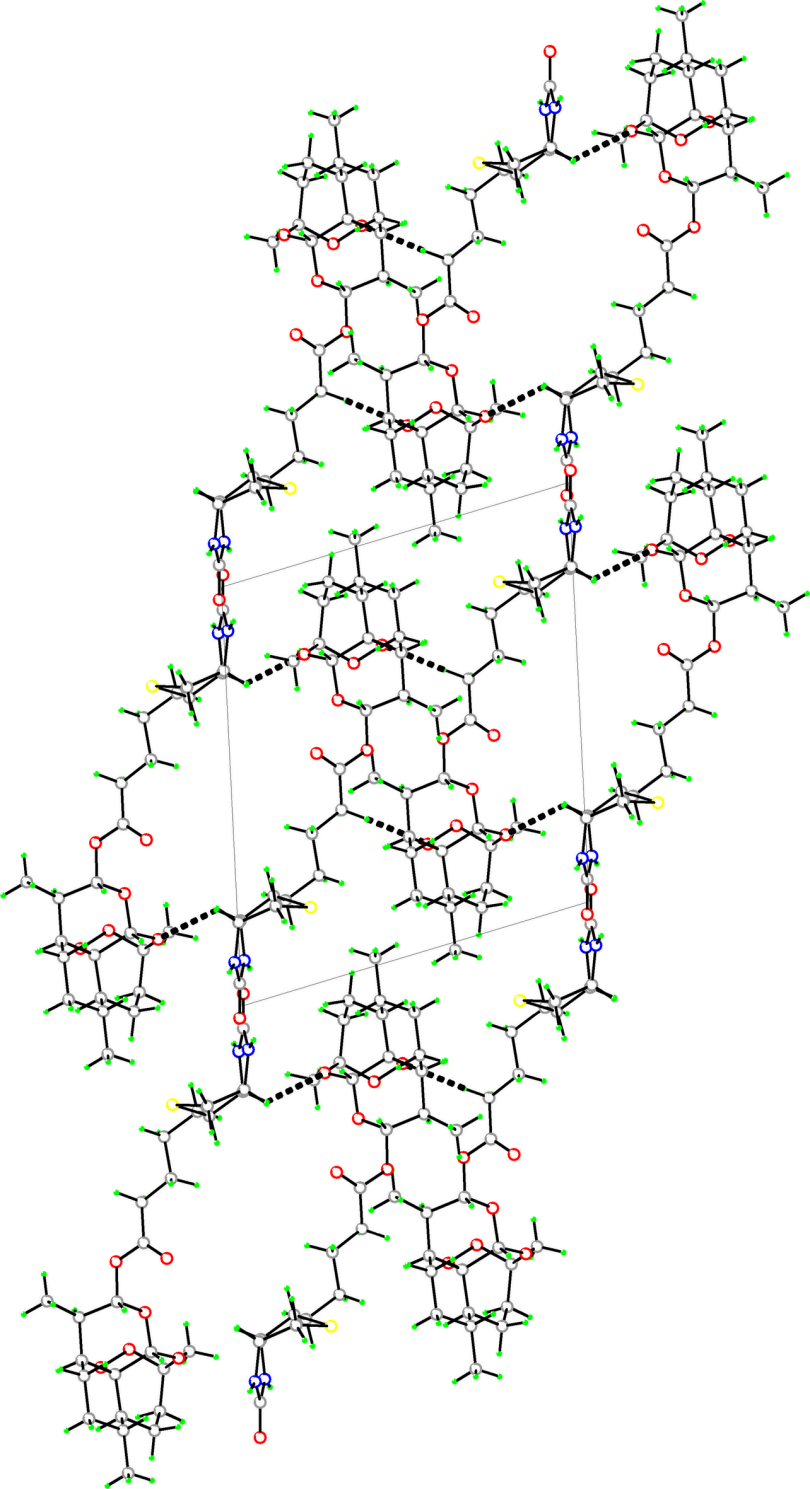
The packing of (**I**) viewed along the *b*-axis direction. Dashed lines indicate C—H⋯O and N—H⋯O hydrogen bonds between adjacent mol­ecules, illustrating the hydrogen-bonded three-dimensional supra­molecular assembly network and the unit-cell arrangement.

The extended structure of (**I**) (Fig. 2 ▸) exhibits a well-defined hydrogen-bonded network dominated by strong N—H⋯O inter­actions between the urea groups of the biotin moieties. These classical amide–urea dimers adopt the characteristic *DADA* double hydrogen-bond motif (Table 1 ▸), a supra­molecular feature commonly observed in biotin and its derivatives: the N1—H1⋯O7 and N2—H2⋯O7 bonds together link adjacent mol­ecules through a pair of nearly linear N—H⋯O hydrogen bonds to generate cyclic dimeric units. Each dimer is stabilized by the anti­parallel orientation of the biotin urea fragments, resulting in a robust ring-like supra­molecular motif.

In addition to these dominant inter­actions, several weaker C—H⋯O hydrogen bonds further consolidate the crystal packing and help maintain the conformation of the di­hydro­artemisinin (DHA) unit and its ester side chain. Notably, a C13—H13*A*⋯O7 contact connects a methyl group of the DHA moiety with the carbonyl oxygen atom of the biotin fragment. A nearly linear (178°) C17—H17*A*⋯O2 inter­action links the methyl­ene group of the linker region to a peroxide oxygen atom within the DHA core, while C24—H24⋯O3 connects the tetra­hydro­thio­phene ring of biotin to an ether oxygen atom of the DHA framework.

These N—H⋯O and C—H⋯O inter­actions inter­link the mol­ecules along the crystallographic *b*-axis through the 2_1_ screw axis, giving rise to a three-dimensional supra­molecular network. Overall, the supra­molecular architecture is primarily governed by the strong dimeric hydrogen bonds between urea groups, complemented by auxiliary C—H⋯O contacts that anchor the flexible DHA skeleton within the lattice while preserving the classical self-recognition mode characteristic of biotin-based systems.

## 4. Database survey

A search of the Cambridge Structural Database (CSD) via the WebCSD inter­face (CSD version 2025.1, May 2025 release; Groom *et al.*, 2016 ▸) for artemisinin-related structures returned 23 hits, predominantly consisting of artemisinin, di­hydro­artemisinin (DHA), artemether, artesunate and their derivatives. Key entries include the parent artemisinin structure with CSD refcode QINGHA (Liu *et al.*, 1979 ▸; Qinghaosu Research Group, 1980 ▸), which confirmed the absolute configuration and endoperoxide bridge essential for anti­malarial activity. The α/β-di­hydro­artemisinin ether dimer YIGGEC (Yue *et al.*, 2006 ▸) and the 7β-hy­droxy­artemisinin derivative GEMBET (Carvalho *et al.*, 2008 ▸) represent metabolically modified analogs generated via microbial transformation. Other notable entries comprise a multicomponent crystal of artesunate with urea aceto­nitrile solvate (CCDC 1590278; Jiang *et al.*, 2020 ▸), illustrating the use of cinchona alkaloids to form multicomponent crystals with artesunate. A trioxane azido derivative LALBON (Xie *et al.*, 2010 ▸) retains the endoperoxide and exhibits weak C—H⋯N/O inter­actions in the solid state. The ferrous bromide rearrangement product of a 5β-hy­droxy-d-secoartemisinin analog (LALBOT; Jahan *et al.*, 2021 ▸) and the corresponding Mosher ester derivative (CCDC 2006194; Jahan *et al.*, 2021 ▸) provide insight into iron-mediated degradation pathways relevant to the mechanism of action. A search for biotin-related small mol­ecules gave 19 hits, including d-biotin (BIOTIN; DeTitta *et al.*, 1976 ▸), de­thio­biotin (DETHIO10; DeTitta & Edmonds, 1980 ▸) and various biotin ester derivatives (*e.g*., BIWYEA; Blauż *et al.*, 2016 ▸). A substructure search for a covalent conjugate featuring both an artemisinin-derived endoperoxide moiety and a biotin-derived ureido­tetra­hydro­thieno[3,4-*d*]imidazole scaffold linked *via* an ester bond, however, returned zero hits. This finding establishes the structural novelty of the present DHA–biotin ester conjugate, whose single-crystal X-ray analysis confirms the retention of the endoperoxide bridge [O1—O2 = 1.462 (4) Å] and the classical N—H⋯O dimeric hydrogen-bonding motif between biotin urea groups, as previously observed in avidin–biotin recognition (Livnah *et al.*, 1993 ▸) and in ferrocene–biotin conjugates (Blauż *et al.*, 2016 ▸).

## 5. Synthesis and crystallization

**Figure 3:**
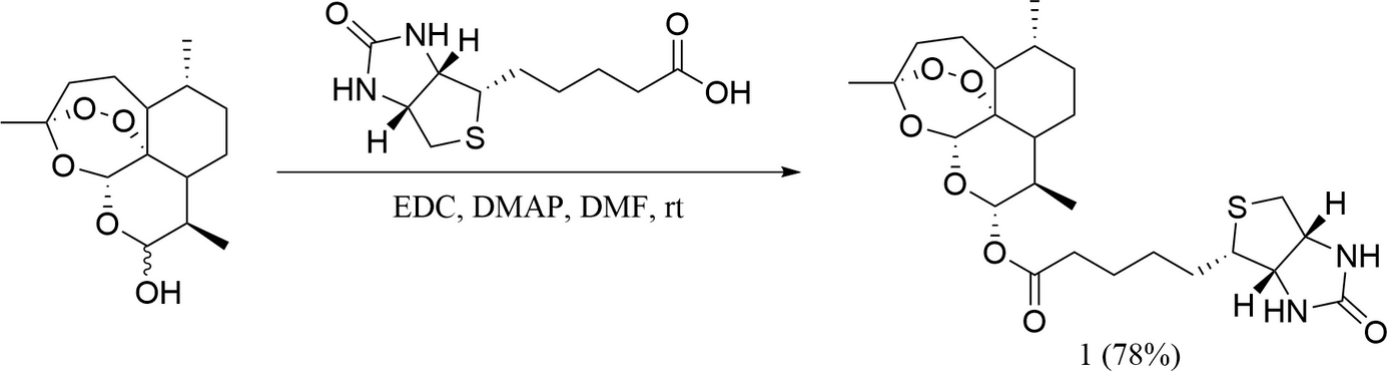
Reaction scheme for obtaining the title compound.

To a solution of biotin (1.0 equiv) and di­hydro­artemisinin (1.1 equiv) in anhydrous di­methyl­formamide (DMF; 2 ml) were added 1-ethyl-3-(3-di­methyl­amino­prop­yl)carbodi­imide (EDC) (3.0 equiv) and 4-di­methyl­amino­pyridine (DMAP) (1.0 equiv) (Fig. 3 ▸) at room temperature under nitro­gen atmosphere. The reaction proceeded smoothly for 2 h to afford the title compound in a yield of 78% (Fig. 3 ▸).

The compound with a purity of over 98% was dissolved in petroleum ether, then left to stand while the solvent was allowed to evaporate gradually under controlled conditions to form colorless needles of (**I**).

## 6. Refinement

Crystal data, data collection and structure refinement details are summarized in Table 2 ▸. There were severely disordered solvent mol­ecules (likely petroleum ether or DMF) in the structure that could not be modeled effectively. Therefore, the SQUEEZE routine (Spek, 2015 ▸) in *PLATON* was used to remove the corresponding electron density. The calculated mol­ecular weight and density do not include the contribution of these squeezed solvents.

## Supplementary Material

Crystal structure: contains datablock(s) global, I. DOI: 10.1107/S2056989026004895/hb8220sup1.cif

Structure factors: contains datablock(s) I. DOI: 10.1107/S2056989026004895/hb8220Isup2.hkl

CCDC reference: 2553196

Additional supporting information:  crystallographic information; 3D view; checkCIF report

## Figures and Tables

**Table 1:** Hydrogen-bond geometry (Å, °)

*D*—H⋯*A*	*D*—H	H⋯*A*	*D*⋯*A*	*D*—H⋯*A*
N1—H1⋯O7^i^	0.88	2.16	3.004 (4)	162
N2—H2⋯O7^ii^	0.88	2.07	2.886 (5)	154
C13—H13*A*⋯O7^iii^	0.98	2.63	3.438 (6)	140
C17—H17*A*⋯O2^iv^	0.99	2.44	3.431 (5)	178
C24—H24⋯O3^v^	1.00	2.44	3.204 (5)	133

Symmetry codes: (i) 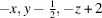; (ii) 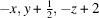; (iii) 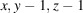; (iv) 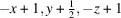; (v) 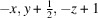.

**Table 2:** Experimental details

Crystal data	
Chemical formula	C_25_H_38_N_2_O_7_S
*M*_r_	510.63
Crystal system, space group	Monoclinic, *P*2_1_
Temperature (K)	150
*a*, *b*, *c* (Å)	12.8909 (8), 7.5857 (4), 14.9027 (9)
β (°)	103.924 (4)
*V* (Å^3^)	1414.46 (15)
*Z*	2
Radiation type	Cu *K*α
μ (mm^−1^)	1.37
Crystal size (mm)	0.08 × 0.03 × 0.01

Data collection	
Diffractometer	Bruker D8 VENTURE DUO PHOTON III
Absorption correction	Multi-scan (*SADABS*; Krause *et al.*, 2015 ▸)
*T*_min_, *T*_max_	0.90, 0.99
No. of measured, independent and observed [*I* > 2σ(*I*)] reflections	16792, 4678, 3874
*R*_int_	0.059
(sin θ/λ)_max_ (Å^−1^)	0.597

Refinement	
*R*[*F*^2^ > 2σ(*F*^2^)], *wR*(*F*^2^), *S*	0.044, 0.123, 1.02
No. of reflections	4678
No. of parameters	320
No. of restraints	1
H-atom treatment	H-atom parameters constrained
Δρ_max_, Δρ_min_ (e Å^−3^)	0.25, −0.23
Absolute structure	Flack *x* determined using 1374 quotients [(*I*^+^)−(*I*^−^)]/[(*I*^+^)+(*I*^−^)] (Parsons *et al.*, 2013 ▸)
Absolute structure parameter	0.034 (14)

Computer programs: *APEX4* and *SAINT* (Bruker, 2021 ▸), *SHELXT2018/2* (Sheldrick, 2015*a* ▸), *SHELXL2018/3* (Sheldrick, 2015*b* ▸) and *OLEX2* (Dolomanov *et al.*, 2009 ▸).

## supplementary crystallographic information

### (3R,5aS,6R,9R,12R,12aR)-3,6,9-Trimethyldecahydro-12H-3,12-epoxy[1,2]dioxepino[4,3-i]isochromen-10-yl 5-((3aS,4S,6aR)-2-oxohexahydro-1H-thieno[3,4-d]imidazol-4-yl)pentanoate Crystal data

**Table:**

C_25_H_38_N_2_O_7_S	*F*(000) = 548
*M**_r_* = 510.63	*D*_x_ = 1.199 Mg m^−^^3^
Monoclinic, *P*2_1_	Cu *K*α radiation, λ = 1.54178 Å
*a* = 12.8909 (8) Å	Cell parameters from 4632 reflections
*b* = 7.5857 (4) Å	θ = 3.1–66.7°
*c* = 14.9027 (9) Å	µ = 1.37 mm^−^^1^
β = 103.924 (4)°	*T* = 150 K
*V* = 1414.46 (15) Å^3^	Needle, colorless
*Z* = 2	0.08 × 0.03 × 0.01 mm

### (3R,5aS,6R,9R,12R,12aR)-3,6,9-Trimethyldecahydro-12H-3,12-epoxy[1,2]dioxepino[4,3-i]isochromen-10-yl 5-((3aS,4S,6aR)-2-oxohexahydro-1H-thieno[3,4-d]imidazol-4-yl)pentanoate Data collection

**Table:**

Bruker D8 VENTURE DUO PHOTON III diffractometer	3874 reflections with *I* > 2σ(*I*)
ω scans	*R*_int_ = 0.059
Absorption correction: multi-scan (SADABS; Krause *et al.*, 2015)	θ_max_ = 66.9°, θ_min_ = 3.1°
*T*_min_ = 0.90, *T*_max_ = 0.99	*h* = −15→15
16792 measured reflections	*k* = −7→9
4678 independent reflections	*l* = −17→17

### (3R,5aS,6R,9R,12R,12aR)-3,6,9-Trimethyldecahydro-12H-3,12-epoxy[1,2]dioxepino[4,3-i]isochromen-10-yl 5-((3aS,4S,6aR)-2-oxohexahydro-1H-thieno[3,4-d]imidazol-4-yl)pentanoate Refinement

**Table:**

Refinement on *F*^2^	H-atom parameters constrained
Least-squares matrix: full	*w* = 1/[σ^2^(*F*_o_^2^) + (0.0757*P*)^2^] where *P* = (*F*_o_^2^ + 2*F*_c_^2^)/3
*R*[*F*^2^ > 2σ(*F*^2^)] = 0.044	(Δ/σ)_max_ = 0.005
*wR*(*F*^2^) = 0.123	Δρ_max_ = 0.25 e Å^−^^3^
*S* = 1.02	Δρ_min_ = −0.23 e Å^−^^3^
4678 reflections	Extinction correction: SHELXL-2018/3 (Sheldrick 2015b), Fc^*^=kFc[1+0.001xFc^2^λ^3^/sin(2θ)]^-1/4^
320 parameters	Extinction coefficient: 0.0052 (9)
1 restraint	Absolute structure: Flack *x* determined using 1374 quotients [(*I*^+^)-(*I*^-^)]/[(*I*^+^)+(*I*^-^)] (Parsons *et al.*, 2013)
Hydrogen site location: inferred from neighbouring sites	Absolute structure parameter: 0.034 (14)

### (3R,5aS,6R,9R,12R,12aR)-3,6,9-Trimethyldecahydro-12H-3,12-epoxy[1,2]dioxepino[4,3-i]isochromen-10-yl 5-((3aS,4S,6aR)-2-oxohexahydro-1H-thieno[3,4-d]imidazol-4-yl)pentanoate Special details

**Table:**

Geometry. All esds (except the esd in the dihedral angle between two l.s. planes) are estimated using the full covariance matrix. The cell esds are taken into account individually in the estimation of esds in distances, angles and torsion angles; correlations between esds in cell parameters are only used when they are defined by crystal symmetry. An approximate (isotropic) treatment of cell esds is used for estimating esds involving l.s. planes.

### (3R,5aS,6R,9R,12R,12aR)-3,6,9-Trimethyldecahydro-12H-3,12-epoxy[1,2]dioxepino[4,3-i]isochromen-10-yl 5-((3aS,4S,6aR)-2-oxohexahydro-1H-thieno[3,4-d]imidazol-4-yl)pentanoate Fractional atomic coordinates and isotropic or equivalent isotropic displacement parameters (Å^2^)

**Table:**

	*x*	*y*	*z*	*U*_iso_*/*U*_eq_	
S1	0.20915 (8)	0.91961 (15)	0.81646 (8)	0.0505 (3)	
O1	0.3657 (2)	−0.0313 (4)	0.2718 (2)	0.0458 (7)	
O2	0.44866 (19)	0.0792 (4)	0.24888 (19)	0.0404 (6)	
O3	0.2270 (2)	0.1621 (4)	0.2161 (2)	0.0456 (7)	
O4	0.3154 (2)	0.2950 (4)	0.34723 (19)	0.0462 (7)	
O5	0.3909 (2)	0.4090 (4)	0.48693 (18)	0.0468 (7)	
O6	0.2471 (3)	0.5877 (5)	0.4587 (2)	0.0628 (9)	
O7	−0.0070 (2)	0.9020 (4)	1.02806 (17)	0.0454 (6)	
N1	0.0146 (3)	0.7662 (4)	0.8946 (2)	0.0361 (7)	
H1	0.027671	0.658762	0.916697	0.043*	
N2	−0.0131 (3)	1.0527 (5)	0.8929 (2)	0.0489 (9)	
H2	−0.029146	1.156896	0.911758	0.059*	
C1	0.2702 (3)	−0.0056 (6)	0.2019 (3)	0.0472 (10)	
C2	0.2875 (4)	−0.0182 (6)	0.1046 (3)	0.0568 (12)	
H2A	0.224701	−0.076423	0.064125	0.068*	
H2B	0.350640	−0.093609	0.106140	0.068*	
C3	0.3047 (4)	0.1613 (7)	0.0624 (3)	0.0530 (11)	
H3A	0.316922	0.140743	0.000095	0.064*	
H3B	0.238135	0.230865	0.054350	0.064*	
C4	0.3979 (3)	0.2722 (6)	0.1184 (3)	0.0440 (10)	
H4	0.464604	0.220533	0.106201	0.053*	
C5	0.3932 (3)	0.4658 (6)	0.0846 (3)	0.0487 (11)	
H5	0.328070	0.521826	0.097374	0.058*	
C6	0.4891 (4)	0.5656 (7)	0.1371 (3)	0.0568 (12)	
H6A	0.484468	0.689124	0.115031	0.068*	
H6B	0.554409	0.512710	0.124660	0.068*	
C7	0.4980 (4)	0.5642 (6)	0.2411 (3)	0.0520 (11)	
H7A	0.435923	0.627083	0.254181	0.062*	
H7B	0.563545	0.627881	0.272931	0.062*	
C8	0.5017 (3)	0.3759 (5)	0.2794 (3)	0.0416 (9)	
H8	0.570105	0.322268	0.272309	0.050*	
C9	0.5033 (3)	0.3668 (5)	0.3833 (3)	0.0428 (9)	
H9	0.515695	0.241411	0.403753	0.051*	
C10	0.3938 (3)	0.4198 (6)	0.3927 (2)	0.0425 (8)	
H10	0.375336	0.541369	0.368037	0.051*	
C11	0.3043 (3)	0.2912 (6)	0.2501 (3)	0.0386 (8)	
H11	0.275535	0.407766	0.224163	0.046*	
C12	0.4096 (3)	0.2584 (5)	0.2232 (3)	0.0375 (9)	
C13	0.1948 (4)	−0.1446 (7)	0.2248 (4)	0.0644 (14)	
H13A	0.120870	−0.113683	0.194582	0.097*	
H13B	0.203215	−0.149263	0.291871	0.097*	
H13C	0.212073	−0.260022	0.202622	0.097*	
C14	0.3851 (4)	0.4786 (8)	−0.0199 (3)	0.0702 (15)	
H14A	0.386812	0.602762	−0.037677	0.105*	
H14B	0.317880	0.425094	−0.053913	0.105*	
H14C	0.445360	0.416090	−0.034798	0.105*	
C15	0.5923 (4)	0.4794 (7)	0.4432 (3)	0.0559 (11)	
H15A	0.597680	0.453881	0.508621	0.084*	
H15B	0.575689	0.604524	0.431167	0.084*	
H15C	0.660379	0.451758	0.428080	0.084*	
C16	0.3104 (3)	0.4963 (6)	0.5113 (3)	0.0464 (10)	
C17	0.3106 (3)	0.4587 (6)	0.6095 (3)	0.0458 (10)	
H17A	0.380400	0.495470	0.649024	0.055*	
H17B	0.304132	0.329780	0.616797	0.055*	
C18	0.2231 (3)	0.5482 (6)	0.6440 (3)	0.0451 (10)	
H18A	0.152821	0.501030	0.610520	0.054*	
H18B	0.223951	0.676273	0.631481	0.054*	
C19	0.2379 (3)	0.5181 (6)	0.7478 (3)	0.0429 (9)	
H19A	0.300397	0.586948	0.781685	0.052*	
H19B	0.253349	0.391870	0.761697	0.052*	
C20	0.1395 (3)	0.5716 (5)	0.7824 (3)	0.0401 (9)	
H20A	0.078893	0.494997	0.752409	0.048*	
H20B	0.154677	0.550105	0.849798	0.048*	
C21	0.1064 (3)	0.7618 (5)	0.7639 (3)	0.0390 (9)	
H21	0.089365	0.780523	0.695482	0.047*	
C22	0.1103 (4)	1.0931 (6)	0.7889 (4)	0.0540 (12)	
H22A	0.132734	1.195311	0.830339	0.065*	
H22B	0.102292	1.132618	0.724264	0.065*	
C23	0.0041 (4)	1.0191 (6)	0.8017 (3)	0.0444 (10)	
H23	−0.056977	1.065423	0.752700	0.053*	
C24	0.0082 (3)	0.8141 (5)	0.7977 (3)	0.0369 (9)	
H24	−0.058346	0.765882	0.755939	0.044*	
C25	−0.0017 (3)	0.9048 (5)	0.9458 (3)	0.0370 (8)	

### (3R,5aS,6R,9R,12R,12aR)-3,6,9-Trimethyldecahydro-12H-3,12-epoxy[1,2]dioxepino[4,3-i]isochromen-10-yl 5-((3aS,4S,6aR)-2-oxohexahydro-1H-thieno[3,4-d]imidazol-4-yl)pentanoate Atomic displacement parameters (Å^2^)

**Table:**

	*U*^11^	*U*^22^	*U*^33^	*U*^12^	*U*^13^	*U*^23^
S1	0.0525 (5)	0.0362 (6)	0.0686 (7)	−0.0132 (5)	0.0258 (5)	−0.0079 (5)
O1	0.0413 (13)	0.0288 (16)	0.0662 (17)	−0.0034 (11)	0.0108 (12)	0.0005 (13)
O2	0.0362 (13)	0.0272 (14)	0.0578 (16)	0.0016 (11)	0.0111 (11)	0.0007 (12)
O3	0.0378 (14)	0.0413 (17)	0.0604 (18)	0.0028 (12)	0.0173 (12)	−0.0038 (14)
O4	0.0519 (15)	0.0422 (17)	0.0504 (16)	0.0006 (13)	0.0237 (12)	−0.0025 (13)
O5	0.0582 (15)	0.0404 (16)	0.0465 (14)	0.0076 (14)	0.0217 (12)	−0.0016 (14)
O6	0.082 (2)	0.060 (2)	0.0547 (18)	0.0288 (19)	0.0323 (16)	0.0079 (17)
O7	0.0724 (17)	0.0240 (14)	0.0471 (15)	0.0008 (13)	0.0285 (12)	0.0000 (13)
N1	0.0512 (18)	0.0223 (17)	0.0400 (18)	−0.0007 (13)	0.0209 (14)	−0.0019 (13)
N2	0.084 (3)	0.0252 (19)	0.046 (2)	0.0104 (17)	0.0325 (18)	0.0021 (15)
C1	0.041 (2)	0.034 (2)	0.065 (3)	0.0002 (17)	0.0097 (18)	−0.004 (2)
C2	0.061 (3)	0.047 (3)	0.060 (3)	−0.003 (2)	0.010 (2)	−0.019 (2)
C3	0.055 (3)	0.056 (3)	0.048 (3)	0.006 (2)	0.011 (2)	−0.008 (2)
C4	0.048 (2)	0.046 (3)	0.041 (2)	0.0097 (18)	0.0172 (17)	0.0022 (19)
C5	0.053 (2)	0.049 (3)	0.049 (2)	0.010 (2)	0.0221 (18)	0.009 (2)
C6	0.059 (3)	0.046 (3)	0.073 (3)	0.005 (2)	0.030 (2)	0.019 (2)
C7	0.061 (3)	0.037 (3)	0.060 (3)	−0.007 (2)	0.019 (2)	0.001 (2)
C8	0.043 (2)	0.034 (2)	0.049 (2)	0.0002 (16)	0.0148 (16)	−0.0025 (18)
C9	0.049 (2)	0.030 (2)	0.050 (2)	0.0015 (16)	0.0136 (17)	−0.0067 (17)
C10	0.057 (2)	0.034 (2)	0.0408 (19)	−0.002 (2)	0.0212 (16)	−0.0039 (19)
C11	0.0414 (19)	0.033 (2)	0.044 (2)	0.0050 (16)	0.0163 (16)	−0.0003 (17)
C12	0.043 (2)	0.028 (2)	0.044 (2)	0.0053 (15)	0.0142 (16)	−0.0028 (16)
C13	0.053 (3)	0.045 (3)	0.091 (4)	−0.010 (2)	0.010 (2)	−0.001 (3)
C14	0.085 (3)	0.076 (4)	0.056 (3)	0.014 (3)	0.030 (2)	0.024 (3)
C15	0.060 (2)	0.053 (3)	0.056 (2)	−0.008 (2)	0.016 (2)	−0.013 (2)
C16	0.056 (2)	0.038 (2)	0.051 (2)	0.012 (2)	0.0242 (19)	0.004 (2)
C17	0.054 (2)	0.041 (3)	0.045 (2)	0.0073 (19)	0.0180 (17)	0.0006 (19)
C18	0.055 (2)	0.041 (2)	0.044 (2)	0.0058 (19)	0.0212 (18)	−0.0022 (19)
C19	0.053 (2)	0.034 (2)	0.047 (2)	0.0064 (17)	0.0240 (17)	0.0061 (18)
C20	0.051 (2)	0.028 (2)	0.048 (2)	0.0020 (17)	0.0229 (17)	−0.0004 (17)
C21	0.047 (2)	0.027 (2)	0.048 (2)	−0.0009 (16)	0.0225 (17)	−0.0023 (17)
C22	0.083 (3)	0.025 (2)	0.066 (3)	−0.001 (2)	0.040 (2)	0.000 (2)
C23	0.065 (3)	0.035 (2)	0.040 (2)	0.0116 (19)	0.0246 (19)	0.0032 (18)
C24	0.043 (2)	0.032 (2)	0.040 (2)	0.0000 (16)	0.0181 (17)	−0.0012 (17)
C25	0.0449 (18)	0.024 (2)	0.046 (2)	0.0011 (16)	0.0177 (15)	0.0004 (18)

### (3R,5aS,6R,9R,12R,12aR)-3,6,9-Trimethyldecahydro-12H-3,12-epoxy[1,2]dioxepino[4,3-i]isochromen-10-yl 5-((3aS,4S,6aR)-2-oxohexahydro-1H-thieno[3,4-d]imidazol-4-yl)pentanoate Geometric parameters (Å, º)

**Table:**

S1—C21	1.817 (4)	C8—H8	1.0000
S1—C22	1.809 (5)	C8—C9	1.545 (6)
O1—O2	1.462 (4)	C8—C12	1.558 (5)
O1—C1	1.422 (5)	C9—H9	1.0000
O2—C12	1.468 (5)	C9—C10	1.506 (5)
O3—C1	1.425 (5)	C9—C15	1.533 (6)
O3—C11	1.402 (5)	C10—H10	1.0000
O4—C10	1.431 (5)	C11—H11	1.0000
O4—C11	1.420 (5)	C11—C12	1.525 (5)
O5—C10	1.416 (4)	C13—H13A	0.9800
O5—C16	1.351 (5)	C13—H13B	0.9800
O6—C16	1.206 (5)	C13—H13C	0.9800
O7—C25	1.245 (4)	C14—H14A	0.9800
N1—H1	0.8800	C14—H14B	0.9800
N1—C24	1.473 (5)	C14—H14C	0.9800
N1—C25	1.344 (5)	C15—H15A	0.9800
N2—H2	0.8800	C15—H15B	0.9800
N2—C23	1.452 (5)	C15—H15C	0.9800
N2—C25	1.358 (5)	C16—C17	1.490 (6)
C1—C2	1.522 (6)	C17—H17A	0.9900
C1—C13	1.527 (6)	C17—H17B	0.9900
C2—H2A	0.9900	C17—C18	1.509 (5)
C2—H2B	0.9900	C18—H18A	0.9900
C2—C3	1.538 (7)	C18—H18B	0.9900
C3—H3A	0.9900	C18—C19	1.529 (5)
C3—H3B	0.9900	C19—H19A	0.9900
C3—C4	1.538 (6)	C19—H19B	0.9900
C4—H4	1.0000	C19—C20	1.534 (5)
C4—C5	1.549 (7)	C20—H20A	0.9900
C4—C12	1.537 (5)	C20—H20B	0.9900
C5—H5	1.0000	C20—C21	1.511 (6)
C5—C6	1.499 (7)	C21—H21	1.0000
C5—C14	1.539 (6)	C21—C24	1.522 (5)
C6—H6A	0.9900	C22—H22A	0.9900
C6—H6B	0.9900	C22—H22B	0.9900
C6—C7	1.526 (6)	C22—C23	1.533 (7)
C7—H7A	0.9900	C23—H23	1.0000
C7—H7B	0.9900	C23—C24	1.558 (5)
C7—C8	1.535 (6)	C24—H24	1.0000
			
C22—S1—C21	88.9 (2)	O2—C12—C4	105.7 (3)
C1—O1—O2	107.6 (3)	O2—C12—C8	102.7 (3)
O1—O2—C12	111.5 (2)	O2—C12—C11	110.7 (3)
C11—O3—C1	114.1 (3)	C4—C12—C8	112.5 (3)
C11—O4—C10	112.5 (3)	C11—C12—C4	112.4 (3)
C16—O5—C10	116.3 (3)	C11—C12—C8	112.1 (3)
C24—N1—H1	123.7	C1—C13—H13A	109.5
C25—N1—H1	123.7	C1—C13—H13B	109.5
C25—N1—C24	112.6 (3)	C1—C13—H13C	109.5
C23—N2—H2	123.9	H13A—C13—H13B	109.5
C25—N2—H2	123.9	H13A—C13—H13C	109.5
C25—N2—C23	112.2 (3)	H13B—C13—H13C	109.5
O1—C1—O3	108.3 (3)	C5—C14—H14A	109.5
O1—C1—C2	113.0 (3)	C5—C14—H14B	109.5
O1—C1—C13	103.1 (4)	C5—C14—H14C	109.5
O3—C1—C2	110.5 (4)	H14A—C14—H14B	109.5
O3—C1—C13	106.9 (4)	H14A—C14—H14C	109.5
C2—C1—C13	114.6 (4)	H14B—C14—H14C	109.5
C1—C2—H2A	108.8	C9—C15—H15A	109.5
C1—C2—H2B	108.8	C9—C15—H15B	109.5
C1—C2—C3	113.8 (4)	C9—C15—H15C	109.5
H2A—C2—H2B	107.7	H15A—C15—H15B	109.5
C3—C2—H2A	108.8	H15A—C15—H15C	109.5
C3—C2—H2B	108.8	H15B—C15—H15C	109.5
C2—C3—H3A	108.4	O5—C16—C17	110.5 (3)
C2—C3—H3B	108.4	O6—C16—O5	123.4 (4)
H3A—C3—H3B	107.4	O6—C16—C17	126.1 (4)
C4—C3—C2	115.6 (4)	C16—C17—H17A	108.5
C4—C3—H3A	108.4	C16—C17—H17B	108.5
C4—C3—H3B	108.4	C16—C17—C18	115.1 (3)
C3—C4—H4	106.3	H17A—C17—H17B	107.5
C3—C4—C5	112.2 (3)	C18—C17—H17A	108.5
C5—C4—H4	106.3	C18—C17—H17B	108.5
C12—C4—C3	112.8 (4)	C17—C18—H18A	109.5
C12—C4—H4	106.3	C17—C18—H18B	109.5
C12—C4—C5	112.5 (3)	C17—C18—C19	110.9 (3)
C4—C5—H5	108.1	H18A—C18—H18B	108.1
C6—C5—C4	110.3 (3)	C19—C18—H18A	109.5
C6—C5—H5	108.1	C19—C18—H18B	109.5
C6—C5—C14	110.1 (4)	C18—C19—H19A	109.0
C14—C5—C4	112.1 (4)	C18—C19—H19B	109.0
C14—C5—H5	108.1	C18—C19—C20	112.8 (3)
C5—C6—H6A	109.2	H19A—C19—H19B	107.8
C5—C6—H6B	109.2	C20—C19—H19A	109.0
C5—C6—C7	112.2 (3)	C20—C19—H19B	109.0
H6A—C6—H6B	107.9	C19—C20—H20A	108.7
C7—C6—H6A	109.2	C19—C20—H20B	108.7
C7—C6—H6B	109.2	H20A—C20—H20B	107.6
C6—C7—H7A	109.2	C21—C20—C19	114.3 (3)
C6—C7—H7B	109.2	C21—C20—H20A	108.7
C6—C7—C8	111.9 (4)	C21—C20—H20B	108.7
H7A—C7—H7B	107.9	S1—C21—H21	107.7
C8—C7—H7A	109.2	C20—C21—S1	113.9 (3)
C8—C7—H7B	109.2	C20—C21—H21	107.7
C7—C8—H8	106.8	C20—C21—C24	114.2 (3)
C7—C8—C9	113.9 (3)	C24—C21—S1	105.3 (3)
C7—C8—C12	112.4 (3)	C24—C21—H21	107.7
C9—C8—H8	106.8	S1—C22—H22A	110.1
C9—C8—C12	109.8 (3)	S1—C22—H22B	110.1
C12—C8—H8	106.8	H22A—C22—H22B	108.5
C8—C9—H9	108.1	C23—C22—S1	107.8 (3)
C10—C9—C8	107.1 (3)	C23—C22—H22A	110.1
C10—C9—H9	108.1	C23—C22—H22B	110.1
C10—C9—C15	112.6 (3)	N2—C23—C22	113.2 (4)
C15—C9—C8	112.7 (3)	N2—C23—H23	110.5
C15—C9—H9	108.1	N2—C23—C24	103.0 (3)
O4—C10—C9	110.5 (3)	C22—C23—H23	110.5
O4—C10—H10	110.9	C22—C23—C24	108.7 (4)
O5—C10—O4	104.3 (3)	C24—C23—H23	110.5
O5—C10—C9	109.0 (3)	N1—C24—C21	113.9 (3)
O5—C10—H10	110.9	N1—C24—C23	101.7 (3)
C9—C10—H10	110.9	N1—C24—H24	110.9
O3—C11—O4	105.7 (3)	C21—C24—C23	108.1 (3)
O3—C11—H11	108.3	C21—C24—H24	110.9
O3—C11—C12	113.0 (3)	C23—C24—H24	110.9
O4—C11—H11	108.3	O7—C25—N1	126.7 (4)
O4—C11—C12	113.1 (3)	O7—C25—N2	124.2 (4)
C12—C11—H11	108.3	N1—C25—N2	109.1 (3)
			
S1—C21—C24—N1	−74.8 (4)	C8—C9—C10—O4	64.4 (4)
S1—C21—C24—C23	37.4 (4)	C8—C9—C10—O5	178.5 (3)
S1—C22—C23—N2	93.4 (4)	C9—C8—C12—O2	−71.1 (4)
S1—C22—C23—C24	−20.5 (5)	C9—C8—C12—C4	175.7 (3)
O1—O2—C12—C4	−108.2 (3)	C9—C8—C12—C11	47.9 (4)
O1—O2—C12—C8	133.7 (3)	C10—O4—C11—O3	179.0 (3)
O1—O2—C12—C11	13.8 (4)	C10—O4—C11—C12	54.8 (4)
O1—C1—C2—C3	−95.4 (4)	C10—O5—C16—O6	−4.2 (7)
O2—O1—C1—O3	−74.8 (3)	C10—O5—C16—C17	174.2 (3)
O2—O1—C1—C2	47.9 (4)	C11—O3—C1—O1	34.9 (4)
O2—O1—C1—C13	172.2 (3)	C11—O3—C1—C2	−89.4 (4)
O3—C1—C2—C3	26.0 (5)	C11—O3—C1—C13	145.3 (4)
O3—C11—C12—O2	−52.7 (4)	C11—O4—C10—O5	178.0 (3)
O3—C11—C12—C4	65.3 (4)	C11—O4—C10—C9	−64.9 (4)
O3—C11—C12—C8	−166.8 (3)	C12—C4—C5—C6	55.2 (4)
O4—C11—C12—O2	67.4 (4)	C12—C4—C5—C14	178.2 (3)
O4—C11—C12—C4	−174.6 (3)	C12—C8—C9—C10	−55.7 (4)
O4—C11—C12—C8	−46.7 (4)	C12—C8—C9—C15	179.9 (3)
O5—C16—C17—C18	−179.4 (4)	C13—C1—C2—C3	146.9 (4)
O6—C16—C17—C18	−1.0 (7)	C14—C5—C6—C7	177.3 (4)
N2—C23—C24—N1	−11.1 (4)	C15—C9—C10—O4	−171.1 (3)
N2—C23—C24—C21	−131.4 (3)	C15—C9—C10—O5	−57.0 (5)
C1—O1—O2—C12	46.5 (4)	C16—O5—C10—O4	−79.5 (4)
C1—O3—C11—O4	−97.3 (4)	C16—O5—C10—C9	162.5 (4)
C1—O3—C11—C12	26.9 (5)	C16—C17—C18—C19	−173.5 (4)
C1—C2—C3—C4	56.9 (5)	C17—C18—C19—C20	−167.7 (4)
C2—C3—C4—C5	−166.2 (3)	C18—C19—C20—C21	−57.8 (5)
C2—C3—C4—C12	−38.0 (5)	C19—C20—C21—S1	−59.4 (4)
C3—C4—C5—C6	−176.5 (3)	C19—C20—C21—C24	179.6 (3)
C3—C4—C5—C14	−53.4 (5)	C20—C21—C24—N1	50.8 (5)
C3—C4—C12—O2	70.6 (4)	C20—C21—C24—C23	163.1 (4)
C3—C4—C12—C8	−178.1 (3)	C21—S1—C22—C23	36.8 (3)
C3—C4—C12—C11	−50.4 (5)	C22—S1—C21—C20	−168.9 (3)
C4—C5—C6—C7	−58.4 (5)	C22—S1—C21—C24	−43.0 (3)
C5—C4—C12—O2	−161.4 (3)	C22—C23—C24—N1	109.3 (4)
C5—C4—C12—C8	−50.0 (4)	C22—C23—C24—C21	−10.9 (5)
C5—C4—C12—C11	77.7 (4)	C23—N2—C25—O7	176.0 (4)
C5—C6—C7—C8	56.9 (5)	C23—N2—C25—N1	−4.6 (5)
C6—C7—C8—C9	−176.3 (3)	C24—N1—C25—O7	175.6 (3)
C6—C7—C8—C12	−50.6 (5)	C24—N1—C25—N2	−3.7 (4)
C7—C8—C9—C10	71.4 (4)	C25—N1—C24—C21	125.6 (4)
C7—C8—C9—C15	−53.0 (5)	C25—N1—C24—C23	9.5 (4)
C7—C8—C12—O2	161.0 (3)	C25—N2—C23—C22	−107.1 (4)
C7—C8—C12—C4	47.8 (4)	C25—N2—C23—C24	10.2 (5)
C7—C8—C12—C11	−80.0 (4)		

### (3R,5aS,6R,9R,12R,12aR)-3,6,9-Trimethyldecahydro-12H-3,12-epoxy[1,2]dioxepino[4,3-i]isochromen-10-yl 5-((3aS,4S,6aR)-2-oxohexahydro-1H-thieno[3,4-d]imidazol-4-yl)pentanoate Hydrogen-bond geometry (Å, º)

**Table:**

*D*—H···*A*	*D*—H	H···*A*	*D*···*A*	*D*—H···*A*
N1—H1···O7^i^	0.88	2.16	3.004 (4)	162
N2—H2···O7^ii^	0.88	2.07	2.886 (5)	154
C13—H13*A*···O7^iii^	0.98	2.63	3.438 (6)	140
C17—H17*A*···O2^iv^	0.99	2.44	3.431 (5)	178
C24—H24···O3^v^	1.00	2.44	3.204 (5)	133

Symmetry codes: (i) −*x*, *y*−1/2, −*z*+2; (ii) −*x*, *y*+1/2, −*z*+2; (iii) *x*, *y*−1, *z*−1; (iv) −*x*+1, *y*+1/2, −*z*+1; (v) −*x*, *y*+1/2, −*z*+1.
